# High Aspartate Aminotransferase/Alanine Aminotransferase Ratio May Be Associated with All-Cause Mortality in the Elderly: A Retrospective Cohort Study Using Artificial Intelligence and Conventional Analysis

**DOI:** 10.3390/healthcare10040674

**Published:** 2022-04-02

**Authors:** Kei Nakajima, Mariko Yuno, Kazumi Tanaka, Teiji Nakamura

**Affiliations:** 1 School of Nutrition and Dietetics, Faculty of Health and Social Services, Kanagawa University of Human Services, 1-10-1 Heisei-cho, Yokosuka 238-8522, Japan; yuno-mariko.5l3@mhlw.go.jp (M.Y.); tanaka-52x@kuhs.ac.jp (K.T.); nakamura-t@kuhs.ac.jp (T.N.); 2 Saitama Medical Center, Department of Endocrinology and Diabetes, Saitama Medical University, 1981 Kamoda, Kawagoe 350-8550, Japan

**Keywords:** alanine aminotransferase, aspartate aminotransferase, AST/ALT ratio, mortality, artificial intelligence, elderly, feature importance, explainable artificial intelligence

## Abstract

Low serum alanine aminotransferase (ALT) activity and high aspartate aminotransferase (AST)/ALT ratio may be associated with high mortality in the elderly. We aimed to confirm this in an 8-year retrospective cohort study. Clinical data for 5958 people living in a city aged 67–104 years were analyzed for their relationships with all-cause mortality using artificial intelligence (AI) and conventional statistical analysis. In total, 1413 (23.7%) participants died during the study. Auto-AI analysis with five rounds of cross-validation showed that AST/ALT ratio was the third-largest contributor to mortality, following age and sex. Serum albumin concentration and body mass index were the fourth- and fifth-largest contributors. However, when serum ALT and AST were individually considered in the same model, the individual serum ALT and AST activities were the seventh- and tenth-largest contributors. Conventional survival analysis showed that ALT, AST, and AST/ALT ratio as continuous variables were all associated with mortality (adjusted hazard ratios (95% confidence intervals): 0.98 (0.97–0.99), 1.02 (1.02–1.03), and 1.46 (1.32–1.62), respectively; all *p* < 0.0001). In conclusion, both AI and conventional analysis suggest that of the conventional biochemical markers, high AST/ALT ratio is most closely associated with all-cause mortality in the elderly.

## 1. Introduction

It can be difficult to identify older members of the community who are at a high risk of mortality, particularly using the conventional biochemical parameters measured in clinical practice. During the past two decades, many studies have shown that low serum alanine aminotransferase (ALT) activity and high aspartate transaminase (AST)/ALT ratio are associated with high mortality in the elderly [1,2,3,4,5,6,7,8,9,10,11,12,13]. However, the mechanisms involved remain to be conclusively established [14,15,16,17]. Some previous studies have also shown that high AST activity is associated with mortality [18,19]. In addition, we previously showed that high serum AST activity, accompanied by normal serum ALT activity, is associated with underweight owing to weight loss [20], which are both risks for mortality.

In clinical practice, the circulating activities of ALT and AST are routinely measured to identify hepatic disease [21,22]. Both transaminases have metabolic functions, in particular in amino acid metabolism, and are expressed in multiple organs, including the liver, myocardium, and skeletal muscle [23,24]. Specifically, ALT is primarily expressed in the liver, whereas AST is widely expressed, including in skeletal muscle. Therefore, a high serum ALT activity reflects the destruction of hepatocytes, whereas a high serum AST activity, alongside a normal ALT activity, may reflect muscle damage. In younger and middle-aged people, long-term increases in serum ALT activity are caused, for example, by non-alcoholic fatty liver disease [25,26], but eventually, the hepatic transaminase activities of the patients may become low during the terminal stage of cirrhosis because of lower ALT synthesis in the liver. In contrast, in the elderly, a low serum ALT activity might be caused by age-related atrophy of the liver or other organs, including the muscles and lungs [27,28]. Given these variations in serum ALT and AST activity, the AST/ALT ratio may more accurately reflect the risk of mortality. Therefore, we performed an 8-year, community-based retrospective cohort study, in which we used both artificial intelligence (AI) and conventional statistical analysis to investigate the relationships of this ratio and other biochemical markers with the risk of mortality. AI techniques, such as machine learning and deep learning, have been shown to improve the prediction of diseases, such as cardiovascular disease [29].

## 2. Materials and Methods

### 2.1. Study Design

We performed an 8-year cohort study of healthcare data relating to people living in Yamato City, a commuter suburb about 30 km from Tokyo [30], in Kanagawa Prefecture. After the conclusion of a contract between Yamato City and Kanagawa University of Human Services, we received the data, which comprised clinical parameters, nursing care level, and mortality but not the cause of death. The study protocol was approved by the ethics committee of Kanagawa University of Human Services (Approval number 17–26), and the study conformed with the principles of the Declaration of Helsinki. The requirement for informed consent was waived because it was a retrospective study of anonymized data. The study protocol was available on the home page of the university [31] and was published in the public relations magazine of Yamato City [32].

### 2.2. Participants and Measurements

Existing data, collected for 6068 people living in Yamato City, Kanagawa Prefecture, who were aged ≥64 years and underwent a baseline examination between April 2011 and March 2012, were reviewed. Owing to incomplete or missing data, 110 individuals were excluded, leaving 5958 for the analysis. Specific inclusion and exclusion criteria were not used, but all the participants had undergone an annual check-up with approval and responded to questions prepared by the medical personnel, which implies that they were not at imminent risk of death, nor did they have severe dementia at baseline. Elderly people who lived in a nursing or retirement home were excluded from the study. In addition, inpatients receiving routine treatments at baseline were also excluded. Clinical measurements were made in the morning after an overnight fast. Body mass index (BMI) was calculated as body mass (kg) divided by the square of height (m^2^). Serum biochemical parameters were measured automatically using standard methods. Estimated glomerular filtration rate (eGFR) was calculated using the equation developed for Japanese populations [33]:

eGFR (mL/min/1.73 m^2^) = 194 × Cr^−1.094^ × Age^−0.287^ (×0.739 if female), where Cr is the serum creatinine concentration (mg/dL).

Questions regarding lifestyle, including smoking habits, alcohol consumption, and pharmacotherapy for hypertension, diabetes, and dyslipidemia, were developed by the municipal officers of Yamato City, referring to the questions prepared by the Japanese Ministry of Health, Labour, and Welfare in 2008 for use in health check-ups in Japan.

### 2.3. Auto-AI Analysis

We performed a graphical user interface (GUI)-based automatic AI (auto-AI) analysis using Prediction One (Sony Network Communications Inc., Tokyo, Japan) [34] and appropriately prepared datasheets. This auto-AI analysis system does not require specific user skills. Data learning, evaluation, and neural network analysis were automatically performed during the analysis to generate an optimal prediction model, with internal cross-validation. The output was a list of parameters, ranked according to the level of contribution of each. Unfortunately, as previously described [35], the details of the AI, including the types of neural network, are trade secrets and therefore not available for publication.

The contributors to mortality were listed in order according to their contribution. The weight of contributor, which is considered as “feature-importance”, is equivalent to the relative contribution calculated using machine learning algorithms of extreme gradient boosting (XGBoost) [34,36]. Feature-importance is one of methods for explainability, which has been considered in the explainable artificial intelligence (XAI) systems [37,38].

For each parameter, the incidences of positive and negative outcomes are shown. After many trials were conducted using the auto-AI analysis, parameters with feature-importance <0.03 were excluded from the contributors. Ten factors (age; sex; smoking status; frequency of alcohol consumption; pharmacotherapy for hypertension, diabetes, and dyslipidemia (yes or no); BMI; white blood cell count; serum albumin concentration; eGFR; the serum AST, ALT, and gamma-glutamyltransferase [GGT] activities; and the AST/ALT ratio) remained as explanatory factors for all-cause mortality.

In the AI analysis, 259 participants whose survival could not be confirmed after 8 years, because they had moved or for other reasons, were excluded. The area under the curve (AUC) for each predictive model was calculated, with >74% and 63–73% being considered to indicate a good and a standard predictive model, respectively [34].

### 2.4. Conventional Statistical Analysis

Data are expressed as mean ± standard deviation (SD) or median (interquartile range). We used the SAS Enterprise Guide (SAS-EG 7.1) in SAS software, version 9.4 (SAS Institute, Cary, NC, USA), which has been used for numerous medical studies worldwide for several decades. Although SAS software also involves an AI system, the procedures involved are complex, particularly for non-SAS users, and require some technical skills, unlike in the case of the auto-AI described above.

The Kaplan–Meier method was used for all-cause mortality analysis. The log-rank and Wilcoxon tests were used to compare participants categorized according to the quartiles of baseline AST/ALT ratio and serum ALT activity. A Cox proportional hazard model, in which the time elapsed until death was considered, was used to calculate the adjusted hazard ratios (HRs) associated with clinical parameters (serum ALT, AST, and AST/ALT ratio; continuous variables). Ten confounding factors were the same factors selected by AI analysis. Conventional statistical analyses were performed using SAS Enterprise Guide (SAS-EG 7.1) in the SAS system, version 9.4 (SAS Institute, Cary, NC, USA). *p* < 0.05 was considered to represent statistical significance

## 3. Results

The baseline characteristics of the participants are shown in Table 1. Although the mean systolic blood pressure of the participants was higher than that of the general Japanese population, the mean values of the other parameters were almost normal [39]. Death occurred in 1413 (23.7%) participants during the 8 years of the study. In addition, a further 1632 (27.4%) participants developed overt disability (long-term care level ≥ 2) during the same period.

Table 2 shows the contributors to all-cause mortality. The AUCs for models 1 and 2 were 73.1% and 72.2%, implying that these were good prediction models.

The AI analysis ranked high AST/ALT ratio as the third-greatest contributor to mortality, following older age and male sex in Model 1. Serum albumin concentration and BMI were the fourth- and fifth-ranked contributors to mortality. The individual serum ALT and AST activities were the seventh- and tenth-greatest contributors in Model 2.

**Figure 1:**
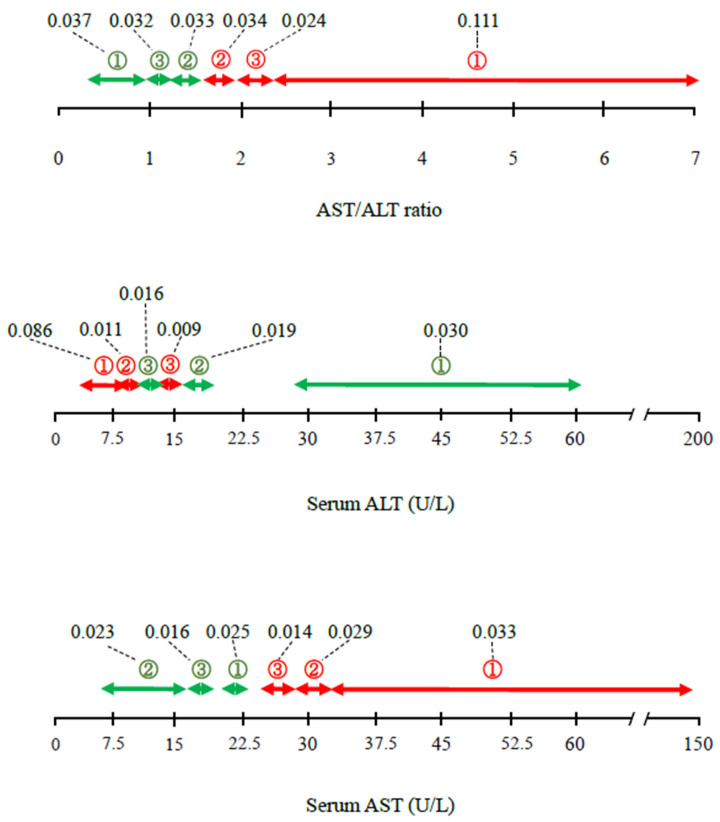
Detailed contributions (ranges and feature-importance) of AST/ALT ratio, ALT activity, and AST activity to the risk of mortality, according to the AI analysis. Values linking to dashed lines express feature-importance, which reflects the contribution degree as a continuous value. Red and green arrows indicate positive and negative contribution to the risk of mortality. ① First-degree contribution; ② second-degree contribution; ③ third-degree contribution. The space in the bar indicates the categories with lower contributions (≥fourth degree). ALT, alanine aminotransferase; AST, aspartate transaminase.

Figure 1 shows the ranges and levels of feature-importance of the AST/ALT ratio and ALT with respect to mortality. AST/ALT ratio ≥ 1.64 (②, ③, ①) was a positive contributor, and AST/ALT ratio ≤ 1.50 (②, ③, ①) was a negative contributor to mortality.

When ALT and AST were considered individually, serum ALT activity ≤ 16 U/L (③, ②, ①) was a positive contributor, and serum ALT ≥ 12 U/L (③, ②, ①) was a negative contributor to mortality, indicating that the ALT ranges associated with each outcome overlap. Serum AST ≥ 25 U/L (③, ②, ①) was a positive contributor, and serum AST ≤ 23 U/L (①, ③, ②) was a negative contributor. Overall, the contributions of the serum ALT and AST activities were smaller than that of AST/ALT ratio. Of all the ranges calculated, the weighting of 0.111 for an AST/ALT ratio of ≥ 2.30 (①) was the largest, and that of 0.086 for an ALT activity of ≤ 10 (①) was the second largest.

**Figure 2:**
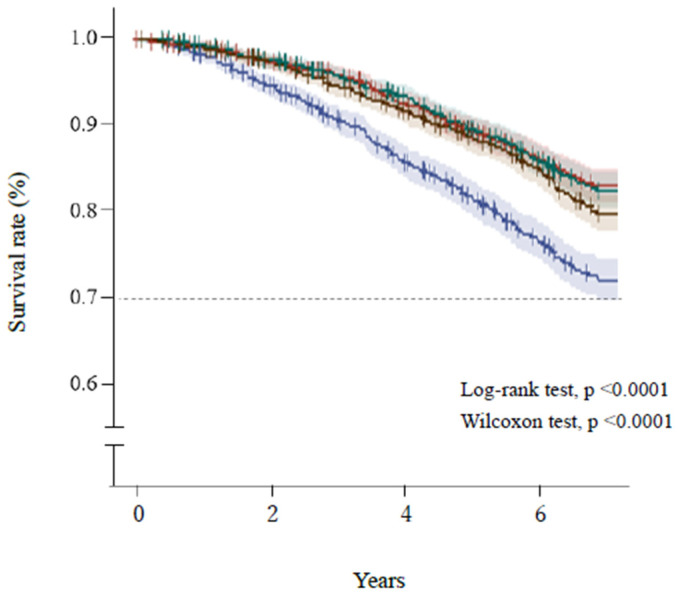
Survival of the participants, categorized according to quartile of AST/ALT ratio. Blue line, ≥1.64 (Q1); brown line, 1.38–1.63 (Q2); green line, 1.15–1.37 (Q3); red line, ≤1.14 (Q4). Short bars express censored cases. The colored areas surrounding the lines show the 95% confidence intervals. ALT, alanine aminotransferase; AST, aspartate transaminase.

**Figure 3:**
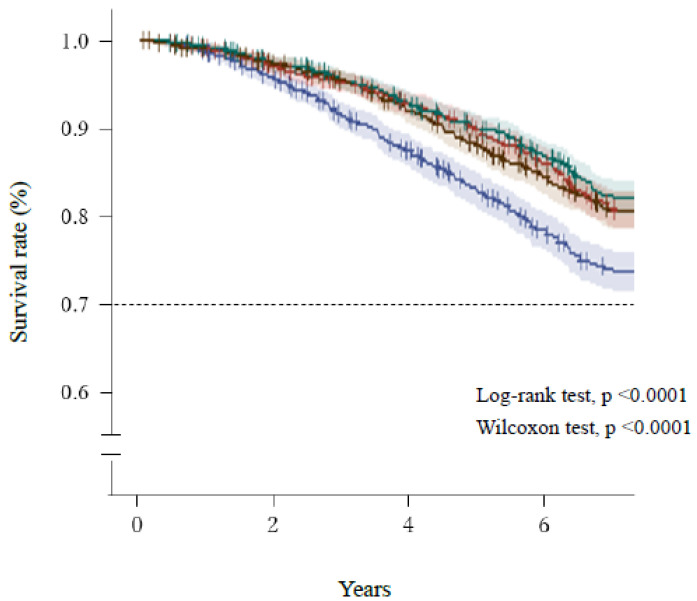
Survival of the participants, categorized according to quartile of serum ALT activity. Blue line, ≤12 U/L (Q1); red line, 13–16 U/L (Q2); green line, 17–21 U/L (Q3); brown, ≥22 U/L (Q4). Short bars express censored cases. The colored areas surrounding the lines show the 95% confidence intervals. ALT, alanine aminotransferase.

Figure 2 and Figure 3 show the Kaplan–Meier survival curves for the mortality of the participants, which were categorized according to quartiles of AST/ALT ratio and serum ALT activity, respectively. Participants with a high AST/ALT ratio and a low ALT activity at baseline were at significantly higher risk of mortality during the study (log-rank and Wilcoxon tests, both *p* < 0.0001). However, Figure 3 shows that after the lowest ALT activity (≤16 U/L, Q1), the highest ALT activity (≥22 U/L, Q4) was also associated with high mortality.

Cox proportional hazard multivariate analysis (Table 3) showed that AST/ALT ratio as a continuous variable was significantly positively associated with all-cause mortality in Model 1. In addition, serum ALT was significantly negatively associated, and AST was significantly positively associated with all-cause mortality in Model 2. Each model was adjusted for the potential confounding factors listed in Table 2. Of note, eGFR was not associated with mortality even though the AI analysis showed eGFR to be the sixth- or seventh-greatest contributor (Table 2).

## 4. Discussion

In the present study, we used auto-AI and conventional analyses to show that a high AST/ALT ratio is more closely associated than low serum ALT activity with all-cause mortality in older community-dwelling Japanese people. The AI showed that older age and male sex, which are unmodifiable factors, are the first- and the second-greatest contributors to the risk of mortality (Table 2), as expected. However, in addition, of the biochemical parameters that are conventionally measured in clinical practice, high AST/ALT ratio was the greatest contributor to the risk of mortality, more so than each individual component; and serum albumin concentration, white blood cell count, and BMI, all of which are well-known risk factors for mortality, also contribute [40,41]. These findings were generated in a single AI analysis, which is a novel approach, but are consistent with previous findings of an association between AST/ALT ratio and high mortality [7,8,10].

As also shown in previous studies [1,2,3,4,5,6,9,11,12], we found that people with low serum ALT activity are at a higher risk of mortality. However, the ranges of serum ALT activity that made positive and negative contributions to mortality overlapped (Figure 1 and Figure 3), which may have reduced the overall contribution calculated for serum ALT activity in the present study. However, the contribution of serum AST to mortality was lower (the tenth-greatest contributor) than those of other parameters, including serum ALT activity alone (seventh-greatest contributor) in the present AI analysis (Table 2). Although many studies have shown associations between low serum ALT activity and mortality [1,2,3,4,5,6,9,11,12], few have shown that high serum AST activity is a risk factor for mortality [18,19]. Therefore, the present findings are consistent with those of the majority of the previous studies concerning serum ALT and AST. Thus, the AST/ALT ratio may be a better predictor of all-cause mortality than the ALT and AST activities alone, and this was confirmed in the present study using both auto-AI and conventional statistical analyses.

Frailty and sarcopenia are strongly associated with multiple adverse clinical outcomes, including death [42,43,44]. However, the identification of these conditions, and particularly of frailty, is time-consuming and labor-intensive compared with the use of blood testing [45,46,47]. In addition, a consensus regarding the definitions of frailty and sarcopenia has not yet been reached [44,48]. Therefore, it is possible that the identification of a useful circulating biomarker may help identify the elderly who are at a high risk of death in the near future.

The normal ranges of serum aminotransferase activities are controversial [49,50]. Therefore, preferably, not only the upper but also the lower limits of normal should be determined for each enzyme in each population. Otherwise, the interpretations of similar results will continue to differ between countries and investigators, leading to conflicting conclusions. In addition, age at least should be considered in the evaluation of serum aminotransferase activities because serum ALT activity likely decreases, and serum AST likely increases with age [51,52,53].

In conventional survival analyses, the weightings of the contributions of each parameter to the outcome are not shown (Table 3), whereas the levels of both positive and negative contributions are indicated until third degrees in the auto-AI system used in the present analysis, which represents its most significant advantage over conventional statistical analyses. Therefore, at present, simultaneous analyses using conventional and AI-based methods may be superior to the use of either alone to confirm and interpret data and to facilitate the most appropriate investigation of the underlying mechanisms even if the results of each differ somewhat.

Meanwhile, a major challenge in AI prediction is that the effectiveness in real-world applications is limited by the inability to explain its decisions [37]. In line with this background, XAI has significantly increased over the last decade [38]. In this study, we considered feature-importance, a method of explainability, which indeed helped us to explain the importance and ranges of clinical permeameters for the risk of mortality.

The present study had some limitations. First, the individual causes of death of the participants were not available. Different results may have been obtained if specific causes of death, for instance, cancer and neurodegenerative diseases, such as Parkinson’s disease and Alzheimer’s disease, had been considered. Second, the time elapsed until death was not considered in the AI analysis because the AUC became very high (92%) when the factor of “time elapsed until death” was included in the predictive model; thus, we excluded this factor from the list of potential contributing factors. Third, we only used a single auto-AI, Prediction One, which is now available in Japan [34], with some limited conditions. Additionally, we did not have an external validation data. Therefore, other AIs, XAI, and different conditions, such as with respect to cross-validation, external validation data, and the adjustment for unbalanced data, may have yielded different results. Fourth, hepatitis B and C virus infection was not measured in this study. However, the prevalences of these infections were reported to be very low (0.6–2.3%) in Japan [54,55] and have declined rapidly [56]. Therefore, the effect of hepatitis virus infection on the mortality may be small if any. Finally, our study consisted of a relatively small sample. Given that machine learning is compatible with large amounts of data, such as big data [57], larger studies that include an AI analysis are thus needed to confirm the present findings.

## 5. Conclusions

In the present study, we used a combination of AI and conventional statistical analysis to show that from among the standard biochemical parameters, high AST/ALT ratio may be best associated with all-cause mortality in older people, following the contributions of age and sex.

## Figures and Tables

**Table 1:** Baseline clinical characteristics of the participants.

N	5958	Normal Range [39]
Men, *n* (%)	3430 (57.6)	
Age (years) Range	79.4 ± 3.9 67–104	
Body mass index (kg/m^2^)	22.7 ± 3.2	18.5–24.9
Systolic blood pressure (mmHg)	132 ± 16.0	<130
Blood parameters		
Alanine aminotransferase (ALT) (IU/L)	16 (13–21)	<31
Aspartate aminotransferase (AST) (IU/L)	22 (19–26)	<31
γ-glutamyl transferase (IU/L)	20 (15–30)	<51
AST/ALT ratio	1.38 (1.15–1.64)	
Triglyceride (mg/dL)	98 (73–134)	<150
High-density lipoprotein-cholesterol (mg/dL)	61 ± 15.8	≥40
HbA1c (%, NGSP)	5.3 ± 0.6	<5.6
Albumin (g/mL)	4.2 ± 0.3	≥4.0
White blood cell (×10^3^, μ/mL)	5.6 ± 1.5	3.2–8.5
History of cardiovascular disease, *n* (%)	877 (14.7)	
Daily alcohol consumption, *n* (%)	1129 (19.0)	
Current smoker, *n* (%)	390 (6.6)	
Having regular exercise, *n* (%)	3185 (53.5)	

Data are presented as mean ± standard deviation, median (interquartile range), or *n* (%).

**Table 2:** Parameters contributing to the prediction of all-cause mortality.

Degree of Contribution (Order)	Model 1		Model 2	
	Variables	Feature-Importance	Variables	Feature-Importance
1	Age (years old)	0.236	Age (years old)	0.229
2	Sex (men, 1; women, 2)	0.119	Sex (men, 1; women, 2)	0.118
3	AST/ALT ratio	0.094	Body mass index (kg/m^2^)	0.094
4	Serum albumin (g/dL)	0.092	Serum albumin (g/dL)	0.094
5	Body mass index (kg/m^2^)	0.089	White blood cell (×10^3^/μL)	0.085
6	White blood cell (×10^3^/μL)	0.083	eGFR (mL/min/1.73 m^2^)	0.073
7	eGFR (mL/min/1.73 m^2^)	0.076	Serum ALT (U/dL)	0.066
8	Serum GGT (U/dL)	0.063	Serum GGT (U/dL)	0.056
9	Current smoking (yes; 1, no; 0)	0.053	Current smoking (yes; 1, no; 0)	0.053
10	Pharmacotherapy for hypertension (yes; 1, no; 0)	0.041	Serum AST (U/dL)	0.049
11	Pharmacotherapy for diabetes (yes; 1, no; 0)	0.038	Pharmacotherapy for hypertension (yes; 1, no; 0)	0.040
12	Pharmacotherapy for dyslipidemia (yes; 1, no; 0)	0.038	Pharmacotherapy for dyslipidemia (yes; 1, no; 0)	0.034
13	Alcohol consumption	0.031	Alcohol consumption	0.033
14			Pharmacotherapy for diabetes (yes; 1, no; 0)	0.030
Total classification accuracy (AUC)	73.1%		72.2%	

Feature importance reflects the contribution degree as a continuous value. In Model 2, the AST and ALT activities were separately included as independent variables. Alcohol consumptions of “almost none”, “sometimes”, or “daily” were coded as 1, 2, and 3, respectively. ALT, alanine aminotransferase; AST, aspartate aminotransferase; GGT, γ-glutamyl transferase.

**Table 3:** Adjusted hazard ratios for all-cause mortality associated with each parameter.

	Variables	Hazard Ratios (95%CIs)	*p*-Value
Model 1			
	Age (years old)	1.12 (1.11–1.13)	<0.0001
	Sex (men, 1; women, 2)	0.48 (0.42–0.54)	<0.0001
	AST/ALT ratio	1.46 (1.32–1.62)	<0.0001
	Serum albumin (g/dL)	0.44 (0.37–0.53)	<0.0001
	Body mass index (kg/m^2^)	0.96 (0.94–0.98)	<0.0001
	White blood cell (×10^3^/μL)	1.06 (1.03–1.10)	0.0001
	eGFR (mL/min/1.73 m^2^)	1.00 (0.99–1.00)	0.250
	Serum GGT (U/dL)	1.00 (1.00–1.01)	<0.0001
	Current smoking (yes; 1, no; 0)	1.31 (1.10–1.57)	0.003
	Pharmacotherapy for hypertension (yes; 1, no; 0)	1.21 (1.08–1.36)	0.001
	Pharmacotherapy for diabetes (yes; 1, no; 0)	1.28 (1.08–1.51)	0.004
	Pharmacotherapy for dyslipidemia (yes; 1, no; 0)	0.88 (0.77–1.00)	0.047
	Alcohol consumption	0.89 (0.83–0.96)	0.002
Model 2			
	Age (years old)	1.12 (1.11–1.14)	<0.0001
	Sex (men, 1; women, 2)	0.49 (0.43–0.55)	<0.0001
	Body mass index (kg/m^2^)	0.95 (0.94–0.97)	<0.0001
	Serum albumin (g/dL)	0.44 (0.36–0.53)	<0.0001
	White blood cell (×10^3^/μL)	1.07 (1.03–1.10)	<0.0001
	eGFR (mL/min/1.73 m^2^)	1.00 (0.99–1.00)	0.123
	Serum ALT (U/dL)	0.98 (0.97–0.99)	<0.0001
	Serum GGT (U/dL)	1.00 (1.00–1.01)	0.0006
	Current smoking (yes; 1, no; 0)	1.37 (1.15–1.64)	0.0005
	Serum AST (U/dL)	1.02 (1.02–1.03)	<0.0001
	Pharmacotherapy for hypertension (yes; 1, no; 0)	1.21 (1.08–1.36)	0.001
	Pharmacotherapy for dyslipidemia (yes; 1, no; 0)	0.87 (0.77–0.99)	0.031
	Alcohol consumption	0.91 (0.84–0.97)	0.007
	Pharmacotherapy for diabetes (yes; 1, no; 0)	1.27 (1.07–1.50)	0.006

AST/ALT ratio, serum albumin, body mass index, white blood cell, eGFR, and serum GGT were enrolled in the multivariate analysis model as continuous (quantitative) variables.

## Data Availability

Not applicable.
